# The College's Attitude to the Bill

**Published:** 1919-03-29

**Authors:** A. Stanley

**Affiliations:** Chairman. The College of Nursing, Ltd., 7 Henrietta Street, Cavendish Square, London, W. 1.


### THE COLLEGE'S ATTITUDE TO THE BILL.

**The following letter has been addressed by the Chairman of the College of Nursing to an inquirer on the subject:—**

DEAR MADAM,—I beg to acknowledge receipt of your letter, in which you ask what attitude the College will take towards the Nurses' Registration Bill, which comes before the House of Commons for second reading on Friday next.

The Council of the College of Nursing has had this matter under its consideration, and it has decided that as the Bill affirms the principle of State recognition of the nursing profession it should, so far, be supported.

I am quite aware, and so are the members of the Council, that a few of the principal supporters of the Nurses' Registration Bill have constantly misrepresented the aims and objects of the College, and have insulted those who were in any way assisting it.

We hold, however, that no personal feeling should be allowed to interfere with the attainment of State Registration, which means so much for nurses, and you will be glad to know that its members are sufficiently broadminded to disregard petty personalities and to work with a single mind for the good of the nursing profession.—I remain, yours faithfully, (Signed) A. STANLEY, Chairman.

The College of Nursing, Ltd., 7 Henrietta Street, Cavendish Square, London, W. 1.

March 25, 1919.

